# Molecular assessment and transcriptome profiling of wild fish populations of *Oryzias mekongensis* and *O*. *songkhramensis* (Adrianichthyidae: Beloniformes) from Thailand

**DOI:** 10.1371/journal.pone.0242382

**Published:** 2020-11-19

**Authors:** Arin Ngamniyom

**Affiliations:** Major in Environment, Faculty of Environmental Culture and Eco-tourism, Srinakharinwirot University, Bangkok, Thailand; National Cheng Kung University, TAIWAN

## Abstract

Among the fish of the genus *Oryzias*, two species are frequently used as model animals in biological research. In Thailand, *Oryzias mekongensis* is usually found in natural freshwater near the Mekong Basin in the northeast region, while *O*. *songkhramensis* inhabits the Songkhram Basin. For differential morphological identification, the coloured bands on the dorsal and ventral margins of the caudal fin are used to distinguish *O*. *mekongensis* from *O*. *songkhramensis*. However, these characteristics are insufficient to justify species differentiation, and little molecular evidence is available to supplement them. This study aimed to investigate the molecular population and transcriptome profiles of adult *O*. *mekongensis* and *O*. *songkhramensis*. In the molecular tree based on cytochrome b sequences, *O*. *mekongensis* exhibited four clades that were clearly distinguished from *O*. *songkhramensis*. Clade 1 of the *O*. *mekongensis* population was close to the Mekong River and lived in the eastern portion of the upper northeast region. Clade 2 was far from the Mekong River and inhabited the middle region of the Songkhram River. Clade 3 was positioned to the west of the Songkhram River, and clade 4 was to the south of the Songkhram River Basin. After RNA sequencing using an Illumina HiSeq 2500 platform, the gene category annotations hardly differentiated the species and were discussed in the text. Based on the present findings, population dispersal of these *Oryzias* species might be associated with geographic variations of the upper northeast region. Molecular genetics and transcriptome profiling might advance our understanding of the evolution of teleost fish.

## Introduction

In the teleost genus *Oryzias*, commonly called “medaka” or “ricefish”, there are more than 25 recognized species in East Asia and Southeast Asia [1, 2]. Two species, Japanese medaka (*O*. *latipes*) and Java medaka (*O*. *javanicus*), have been utilized as nonmammalian vertebrate models in many molecular biology studies [3, 4]. Experiments using medaka have been performed in fields including molecular physiology, endocrinology, genetics and evolution [3, 5–7]. In Thailand, five species of *Oryzias* have been reported: *O*. *minutillus*, *O*. *javanicus*, *O*. *dancena*, *O*. *mekongensis* and *O*. *songkhramensis* [1, 8, 9]. *Oryzias minutillus* is found throughout freshwater environments in all regions [10], while *O*. *javanicus* and *O*. *dancena* have a wide range in brackish water [1].

In the northeast region of Thailand, *O*. *mekongensis* is widely found in natural freshwater, such as paddy fields and shallow ponds, in the Mekong River Basin, while *O*. *songkhramensis* is commonly found in the middle area of the upper northeast region based on the Songkhram River Basin [8, 11]. For morphological identification, orange-red and black bands on the dorsal and ventral portions of the caudal fin are predominant in *O*. *mekongensis* [11]. In contrast, light yellow and thin black bands appear on the dorsal and ventral margins of the caudal fin of *O*. *songkhramensis* [8]. However, these characteristics are too obscure or insufficient to justify the differentiation of *O*. *mekongensis* from *O*. *songkhramensis* in the overlapping habitats of both species [12].

Cytochrome b (cytb) is a mitochondrial DNA-encoded polypeptide that has been used for molecular genetic variation in several studies of bony fish [13–15]. Takehana et al. [16] previously clarified the natural distribution of wild Japanese medaka in association with geographic variations based on the cytochrome b gene. The cytb gene is also a potential molecular genetics tool to evaluate fish populations, including medaka [13–16]. In Thailand, only *O*. *minutillus* has been investigated at the population genetics level in association with geographic variation [12]. In contrast, the genetic diversity of *O*. *mekongensis* and *O*. *songkhramensis* in the northeast region of Thailand remains unclear [12].

Transcriptome sequencing is an approach for the analysis of mRNAs that allows the investigation of a set of RNA transcripts to obtain information about gene prediction, gene pathways and gene function [17, 18]. This approach has provided profiles that improved our understanding of molecular processes in many organisms [19–22].

RNA sequencing (RNA-Seq) has been used to achieve transcriptome profiling in teleost fish research [23]. For instance, previous works included transcriptome analysis of Selincuo naked carp (*Gymnocypris selincuoensis*) [24], comparative transcriptome analysis of four Percidae [25] and transcriptomic characterization of the response to ectoparasitic infection in mangrove rivulus (*Kryptolebias marmoratus*) [26]. In fish species of *Oryzias*, transcriptome sequencing data sets have been reported for *O*. *javanicus*, *O*. *melastigma*, *O*. *latipes* and *O*. *woworae* [3, 27–30]. Recently, Ngamniyom et al. [12] provided transcriptome profiling data for male and female *O*. *minutillus*.

Therefore, the aims of this study were to investigate the molecular populations and transcriptome profiles of wild adult fish of *O*. *mekongensis* and *O*. *songkhramensis* from Thailand. Furthermore, we emphasized the RNA-Seq data of both species of *Oryzias* to improve the genetic resources for freshwater teleosts.

## Materials and methods

**Fig 1:**
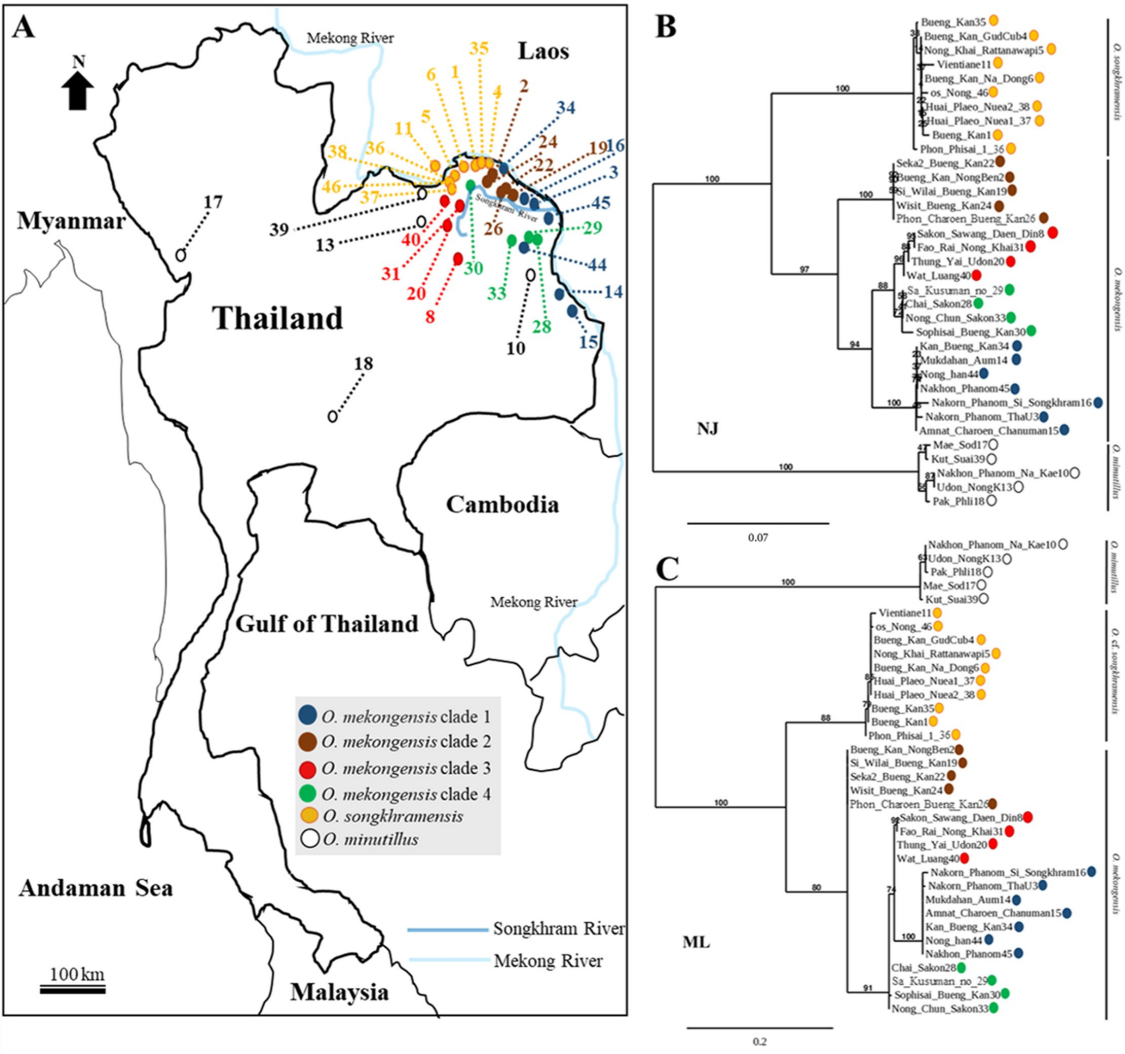
Map drawing of fish collections in the northeast region of Thailand. (A) Neighbour joining (NJ) method (B) and maximum likelihood (ML) method (C) for the molecular trees of *O*. *mekongensis* and *O*. *songkhramensis* based on the mitochondrial cytb gene. Brown, red, blue and green circles indicate the individual sample of *O*. *mekongensis* for each clade. Yellow circles represent the samples of *O*. *songkhramensis*. White circles indicate *O*. *minutillus* as an outgroup.

Fish were randomly captured from shallow ponds, ditches and paddy fields in the northeast region of Thailand by using a hand net (Fig 1A) and (Table 1). All field sites for fish sampling were in public areas that did not require any special permit. To confirm the species of *Oryzias*, adult fish individuals with a standard length (>18 mm) were anaesthetized with 100 mg/L tricaine methanesulfonate solution (MS-222) (Sigma-Aldrich, MO, USA). After anaesthesia, *O*. *mekongensis* and *O*. *songkhramensis* were identified according to the key ricefish species in Thailand described by Magtoon [8] and Termvidchakorna and Magtoon [9]. The caudal fins of *O*. *mekongensis* and *O*. *songkhramensis* were dissected from the fish bodies and stored in absolute ethanol at −20°C prior to DNA extraction. The procedure used for the sacrifice of fish was consistent with the Canadian Council on Animal Care Guidelines on the Care and Use of Fish in Research, Teaching and Testing, 2005 (https://www.ccac.ca/Documents/Standards/Guidelines/Fish.pdf) and the National and Institutional Guidelines for Animal Care and Use for Vertebrates from the Institute for Animals for Scientific Purpose Development (IAD) National Research Council of Thailand (NRCT). The Animal Care and Use Committee of Srinakharinwirot University provided ethics approval for all animal experiments in this study, and the approval license was SWU-A-026_2562.

**Table 1:** Geographic coordinates of collection sites and accession numbers of cytb sequences.

specimen	geographic coordinate	species	accession number
Bueng Kan1	18°23'45.2"N 103°25'58.4"E	*O*. *songkhramensis*	MN657279
Bueng Kan35	18°24'29.2"N 103°27'40.4"E	*O*. *songkhramensis*	MN657280
NongBen2	18°20'34.1"N 103°39'50.4"E	*O*. *mekongensis*	MN657281
Nakorn Phanom ThaU3	17°38'22.0"N 104°26'02.5"E	*O*. *mekongensis*	MN657282
GudChap4	18°24'00.6"N 103°32'48.9"E	*O*. *songkhramensis*	MN657283
Nong Khai Rattanawapi5	18°14'20.8"N 103°11'13.6"E	*O*. *songkhramensis*	MN657284
Na Dong6	18°16'11.2"N 103°17'32.1"E	*O*. *songkhramensis*	MN657285
Sawang Daen Din8	17°24'00.3"N 103°21'25.5"E	*O*. *mekongensis*	MN657286
Na Kae10	16°57'45.8"N 104°27'48.8"E	*O*. *minutillus*	MN657287
Vientiane11	18°12'17.0"N 102°49'31.8"E	*O*. *songkhramensis*	MN657288
Udon NongK13	17°43'04.3"N 102°50'50.8"E	*O*. *minutillus*	MN657289
Mukdahan Aum14	16°21'55.5"N 104°33'24.8"E	*O*. *mekongensis*	MN657290
Chanuman15	16°13'48.3"N 104°59'09.7"E	*O*. *mekongensis*	MN657291
Si Songkhram16	17°43'12.0"N 104°19'06.2"E	*O*. *mekongensis*	MN657292
Mae Sod17	16°42'27.2"N 98°36'43.8"E	*O*. *minutillus*	MN657293
Pak Phli18	14°05'37.1"N 101°16'37.0"E	*O*. *minutillus*	MN657294
Si Wilai19	17°57'31.1"N 104°02'15.5"E	*O*. *mekongensis*	MN657295
Thung Yai20	17°30'24.8"N 103°12'03.2"E	*O*. *mekongensis*	MN657296
Seka22	18°11'46.3"N 103°44'57.1"E	*O*. *mekongensis*	MN657297
Wisit24	18°22'17.0"N 103°37'08.8"E	*O*. *mekongensis*	MN657298
Phon Charoen26	18°01'32.0"N 103°37'31.1"E	*O*. *mekongensis*	MN657299
Chai Sakon28	17°20'48.2"N 104°15'54.7"E	*O*. *mekongensis*	MN657300
SKusuman29	17°21'47.7"N 104°10'29.5"E	*O*. *mekongensis*	MN657301
Sophisai30	18°08'25.4"N 103°31'03.0"E	*O*. *mekongensis*	MN657302
Fao Rai31	17°59'30.5"N 103°23'16.4"E	*O*. *mekongensis*	MN657303
Nong Chun33	17°35'46.7"N 104°00'23.4"E	*O*. *mekongensis*	MN657304
Kan Bueng Kan34	18°15'24.6"N 103°52'10.9"E	*O*. *mekongensis*	MN657305
Phon Phisai36	18°05'18.2"N 103°04'57.7"E	*O*. *songkhramensis*	MN657306
Huai Plaeo Nuea37	18°03'06.7"N 103°08'07.3"E	*O*. *songkhramensis*	MN657307
Huai Plaeo Nuea38	18°03'41.0"N 103°08'56.4"E	*O*. *songkhramensis*	MN657308
Kut Suai39	17°54'19.6"N 103°03'12.3"E	*O*. *minutillus*	MN657309
Wat Luang40	17°55'25.3"N 103°03'28.6"E	*O*. *mekongensis*	MN657310
Nong han44	17°15'39.3"N 104°09'16.8"E	*O*. *mekongensis*	MN657311
Nakhon Phanom45	17°36'42.3"N 104°26'12.1"E	*O*. *mekongensis*	MN657312
os Nong46	18°03'26.9"N 103°05'25.4"E	*O*. *songkhramensis*	MN657313

The genomic DNA of the medaka fin was extracted by using the DNeasy Blood & Tissue Kit (Qiagen, Germany) according to the manufacturer's protocol. The paired primers used for DNA target amplification of cytb were 5´–ggACgCTCCgCTgCTAgCCC–3´ and 5´–CCTggTTTgggAgTCAggg–3´ (~1394 bp) with Ex *Taq* polymerase (Takara, Japan). The PCR thermal cycling steps consisted of an initial denaturation at 95°C for 3 min; 33 cycles of denaturation for 30 s at 94°C, annealing for 40 s at 55°C, and extension for 2 min at 72°C; and a final extension for 5 min at 72°C. The PCR products were confirmed on 1% agarose gels stained with SmartGlow™ Safe Green Pre-Stain (Accuris Instruments, US) under a Blue light DNA transilluminator, and they were extracted from the gels by using the QIAquick Gel Extraction Kit (Qiagen, Germany) according to the manufacturer's instructions. DNA sequencing was conducted using an automated DNA analyser ABI 3730xl system (Applied Biosystems, US). Nucleotide sequences were deposited in GenBank from the National Center for Biotechnology Information (NCBI) with the following accession numbers: MN657279-MN657313 for cytb. The nucleotide sequences of all samples were aligned and trimmed with MUltiple Sequence Comparison by Log-Expectation (MUSCLE) software [31] and curated using Gblocks [32]. Molecular trees with neighbour-joining and maximum likelihood were generated and rendered using PhyML with 1000 bootstraps and TreeDyn [33]. Bootstrap support was justified at > 70%.

For RNA-Seq analysis, twenty males and twenty females of *O*. *mekongensis* (n = 40) sampled evenly from all sites and twenty males and twenty females of *O*. *songkhramensis* (n = 40) sampled equally from all sites were separated by sex and species and maintained in aquariums containing freshwater without chlorine. The environments of the aquaria were established, and the fish were allowed to acclimatize for 1 week under the following conditions: pH, 6.9–7.3; salinity, 0.05–0.06 ppt; 27–29°C; dissolved oxygen, 6.0–6.5 mg/L. The fish were fed *ad libitum* 2 times per day with Kyorin Hikari food for medaka (Kyorin Food Industries, Hyogo, Japan) under a photoperiod of 12 hr light:12 hr darkness. The water in the fish aquaria was changed every day.

Total RNA extractions were performed by using an RNeasy Mini Kit (Qiagen, Germany) according to the manufacturer's instructions. During RNA purification, the nucleic solutions were treated with RNase-free DNase I (Qiagen, Germany). The quality of the total RNA was evaluated by gel electrophoresis in 1% agarose gels. The concentrations were quantified by using Thermo Scientific™ NanoDrop 2000 and 2000c (Thermo Fisher Scientific, MA, USA) and confirmed via Qubit 2.0 fluorometric quantitation (Thermo Fisher Scientific, MA, USA). Equal concentrations of RNA of both species were preserved at –80°C.

After the quality control procedures, the mRNAs were enriched from the total RNA using oligo(dT)_25_ beads (200 ng RNA per sample), and fragments were randomly placed in RNA fragmentation buffer (Illumina, CA, USA). The rRNA was removed using the Illumina Ribo-Zero Plus rRNA Depletion Kit (Illumina, WI, USA). For first-strand synthesis, complementary DNA (cDNA) synthesis was performed using a TruSeq mRNA kit (Illumina, CA, USA) with random hexamers and a SuperScript® III First-Strand Synthesis System (Invitrogen, CA, USA). Second-strand cDNA was synthesized in a buffer (mRNAseq Illumina, CA, USA) supplemented with *E*. *coli* polymerase I, RNase H and dNTPs, and the cDNAs were purified by AMPure XP beads. The cDNA library quantification was measured by using a Qubit 2.0 fluorometer, and the insert size was verified with an Agilent 2100 Bioanalyzer with an Agilent RNA 6000 Nano Kit (Agilent Technologies, MD, USA). The sequencing was processed on a HiSeq 2500 Sequencing System (Illumina, CA, USA) according to Dillies et al. [34]. The accession numbers of the transcriptome data were GSE142602, GPL27950, GSM4232740, PRJNA635451 and SAMN15041390, which were deposited in a public functional genomics database, the Gene Expression Omnibus (GEO) (https://www.ncbi.nlm.nih.gov/geo/) and Sequence Read Archive (SRA) database (https://www.ncbi.nlm.nih.gov/sra). For transcriptome reconstruction, SOAPnuke v1.5.2 software was used to filter raw reads, low-quality reads, noisy reads, adaptor-polluted reads and reads with a high content of unknown bases to obtain clean reads. Trinity software (r20140413p1) composed of Inchworm, Chrysalis and Butterfly was used to analyse the assembly data, and Tgicl v2.0.6 was applied to cluster transcripts [35]. Nt (nucleotide) databases were annotated by using NCBI blast v2.2.23. Diamond software v0.8.31 was used for Nr (NCBI non-redundant protein sequences), KOG (euKaryotic Orthologous Groups) and KAAS r140224 for KEGG (Kyoto Encyclopedia of Genes and Genome). Pfam and protein prediction were analysed by the HMMER 3.0 package hmmscan and Blast2GO v2.5.0. NR was used to carry out Gene Ontology (GO) annotation. KEGG annotations were assigned by using the KEGG Automatic Annotation Server. KEGG enrichment was performed with GOSeq 1.10.0, topGO 2.10.0 for GO and KOBAS v2.0.12 for KEGG. SwissProt (UniProtKB/Swiss-Prot) was used for the manually annotated and reviewed database of the UniProt Knowledgebase (UniProtKB) (http://ftp.ebi.ac.uk/pub/databases/swissprot). Unigenes encoding predicted transcription factors (TFs) were mapped to the AnimalTFDB2.0 database to obtain the TF family using getorf EMBOSS:6.5.7.0 with–minsize 150 parameters and hmmsearch v3.0. Differentially expressed genes (DEGs) were identified according to the gene expression levels of both groups by using DEGseq, DEseq2, EBseq, NOIseq and PossionDis to detect the DEGs. The DEG identification methods used the log2-fold change ratios between samples and the P value (cut off 0.05) and q-value for PossionDis or DEseq2. Heatmaps of TF expression and DEGs were created with pheatmap and the appropriate function of R.

## Results

The *O*. *songkhramensis* populations were found to be limited to the upper west part of the Songkhram River Basin, while the *O*. *mekongensis* populations were spread throughout the upper northeast region based on the Mekong River Basin (Fig 1A). Regarding molecular variation based on the cytb gene, *O*. *mekongensis* (n = 20) was clearly isolated from *O*. *songkhramensis* (n = 10) with bootstrap support (≥ 80%) and from *O*. *minutillus* (n = 5) (≥ 100%) for the neighbour-joining and maximum likelihood methods (Fig 1B and 1C).

All specimens of *O*. *mekongensis* were monophyletic and were a sister group to the *O*. *songkhramensis* populations. Among the three *Oryzias* species, the *O*. *mekongensis* populations were more closely related to *O*. *songkhramensis* than were the *O*. *minutillus* population. Four clades of wild fish populations were represented at the molecular level in *O*. *mekongensis* according to neighbour-joining and maximum likelihood (≥ 70%). In contrast, there were no subgroups of the *O*. *songkhramensis* population, which had only a single clade. For the clades of the *O*. *mekongensis* population, clade 1 approached the Mekong River and lived in the eastern part of the upper northeast region (n = 7). The fish in clade 2 spread out from the Mekong River and were distributed across the middle part of the Songkhram River (n = 5). Clade 3 of the *O*. *mekongensis* population naturally inhabited the western part of the Songkhram River Basin (n = 4). Conversely, clade 4 comprised the fish population in the southern part of the Songkhram River (n = 3). However, one sample of this clade was biased to the west (Fig 1B and 1C).

**Fig 2:**
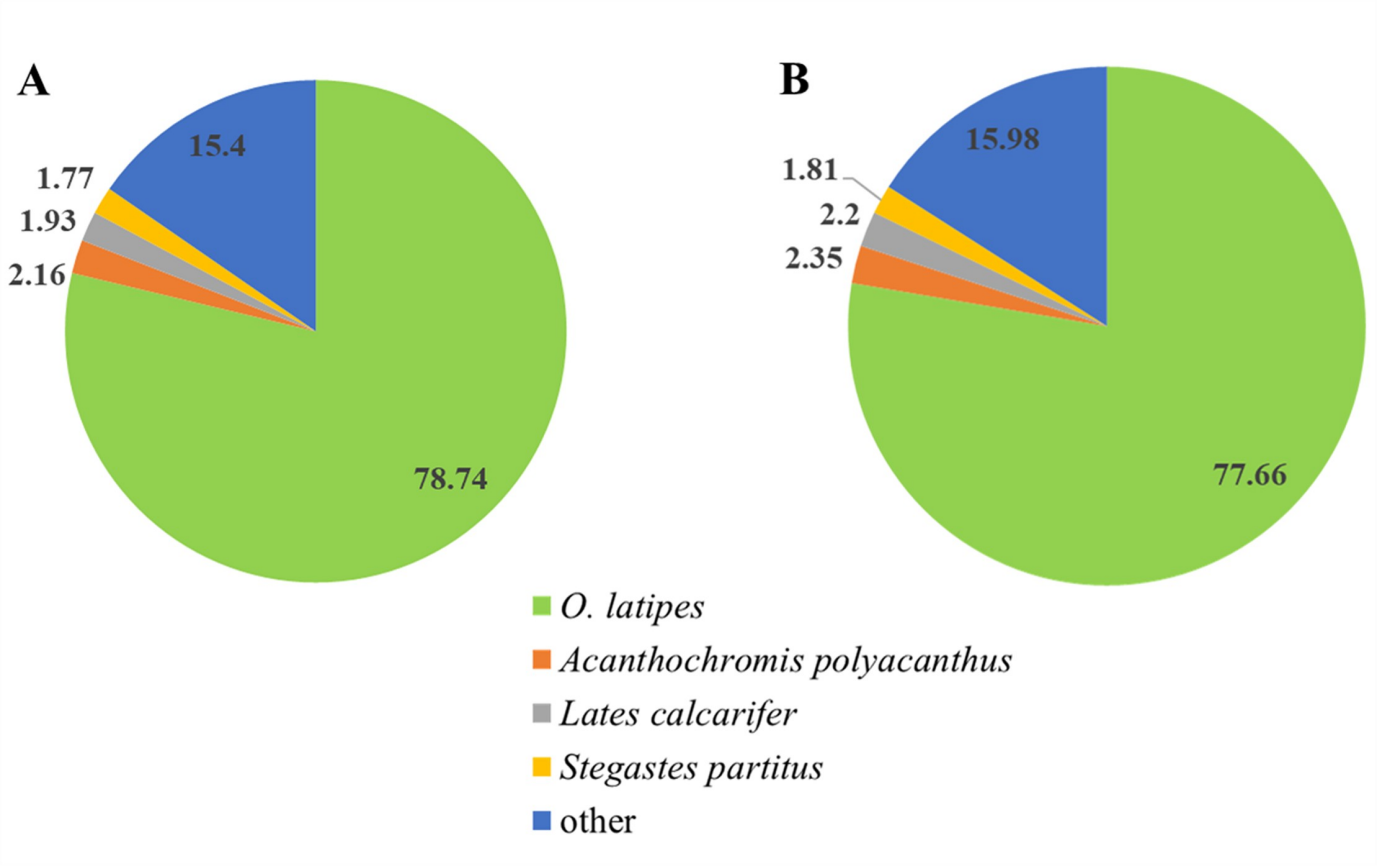
Distribution of non-redundant database annotated species (%) of *O*. *Mekongensis* (A) and *O*. *Songkhramensis* (B). The image refers to *O*. *latipes*, *A*. *polyacanthus*, *L*. *calcarifer* and *S*. *partitus*.

**Fig 3:**
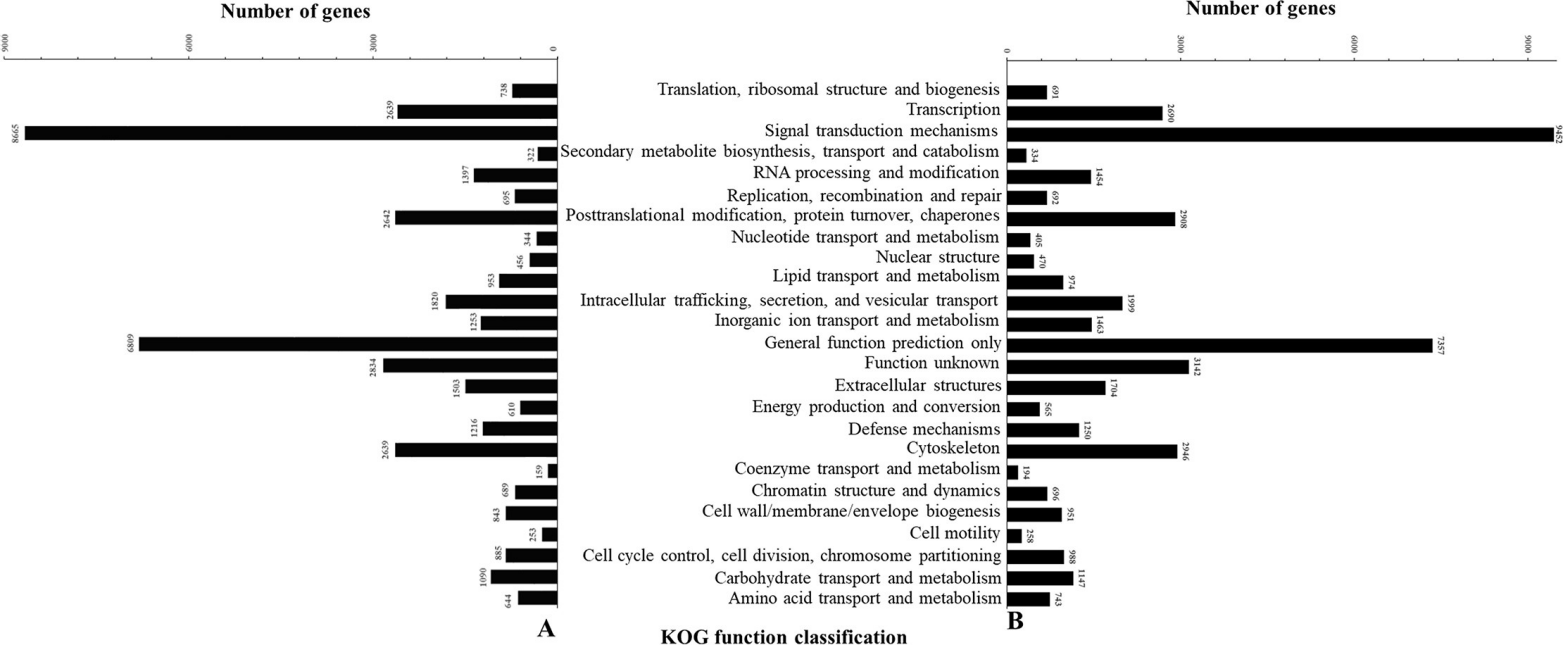
EuKaryotic Orthologous Groups (KOG) classification and gene number in *O*. *Mekongensis* (A) and *O*. *Songkhramensis* (B). Twenty-five classes of KOG were observed between the two species.

**Fig 4:**
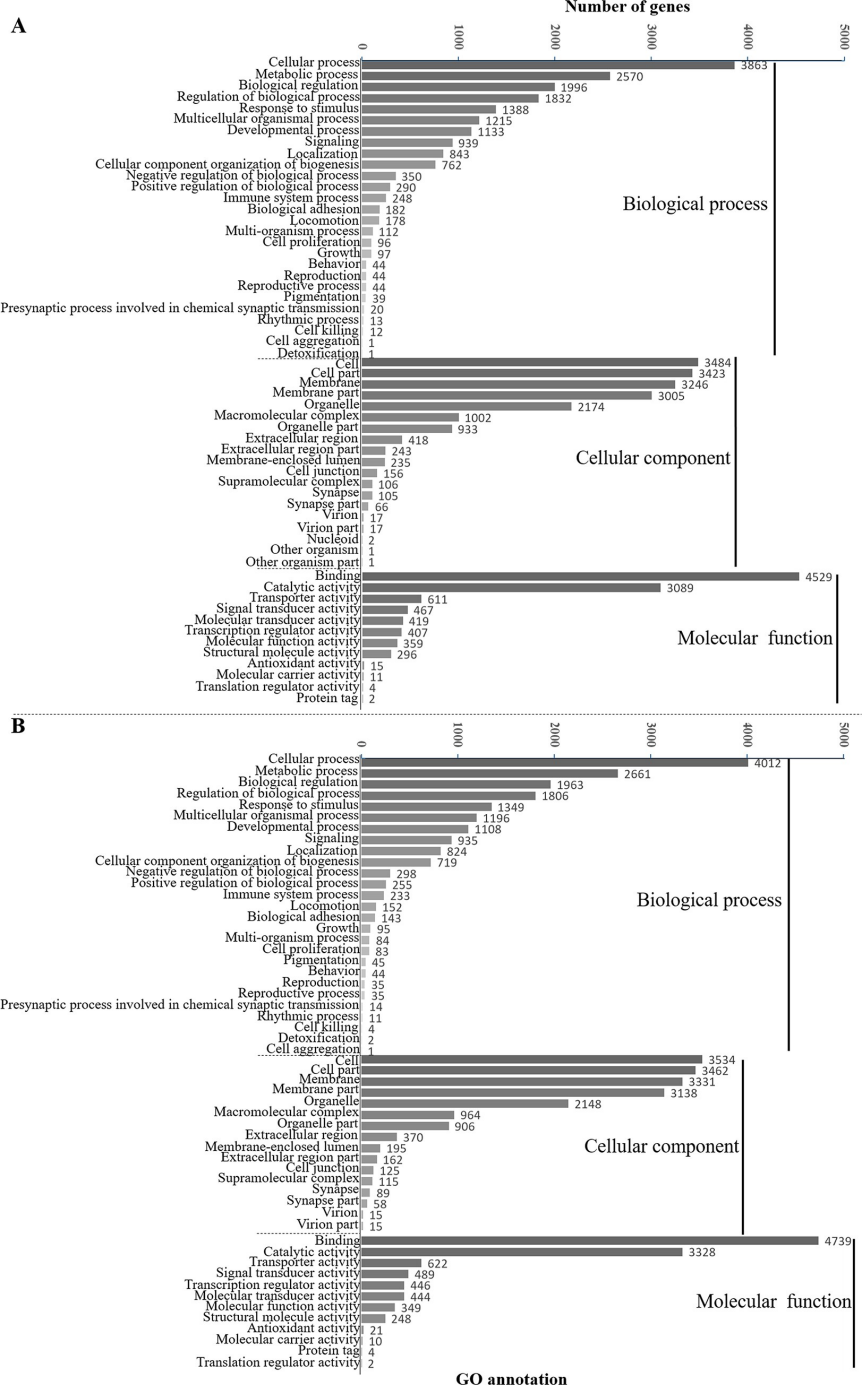
Gene Ontology (GO) for annotation of gene number in *O*. *Mekongensis* (A) and *O*. *Songkhramensis* (B). Three major criteria for GO between both species.

**Fig 5:**
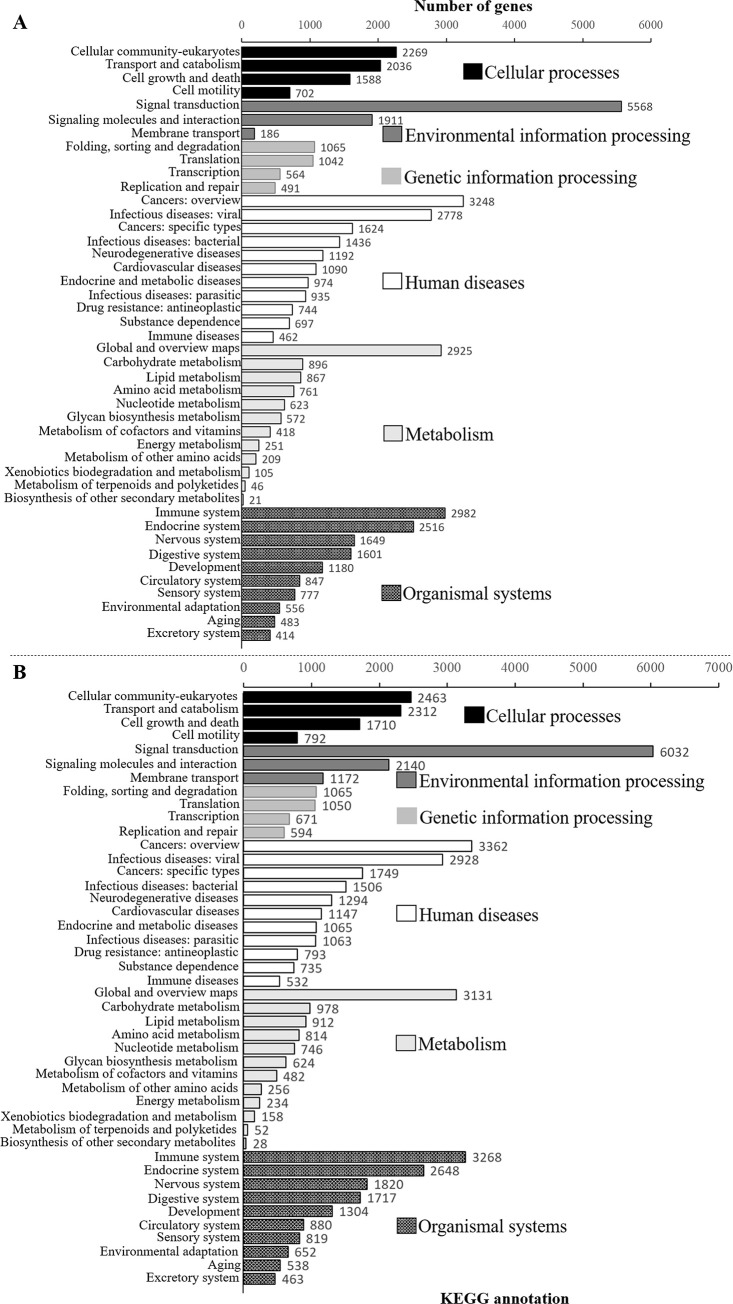
Kyoto Encyclopedia of Genes and Genomes (KEGG) in *O*. *Mekongensis* (A) and *O*. *Songkhramensis* (B). They represented the six main classifications and gene numbers.

**Fig 6:**
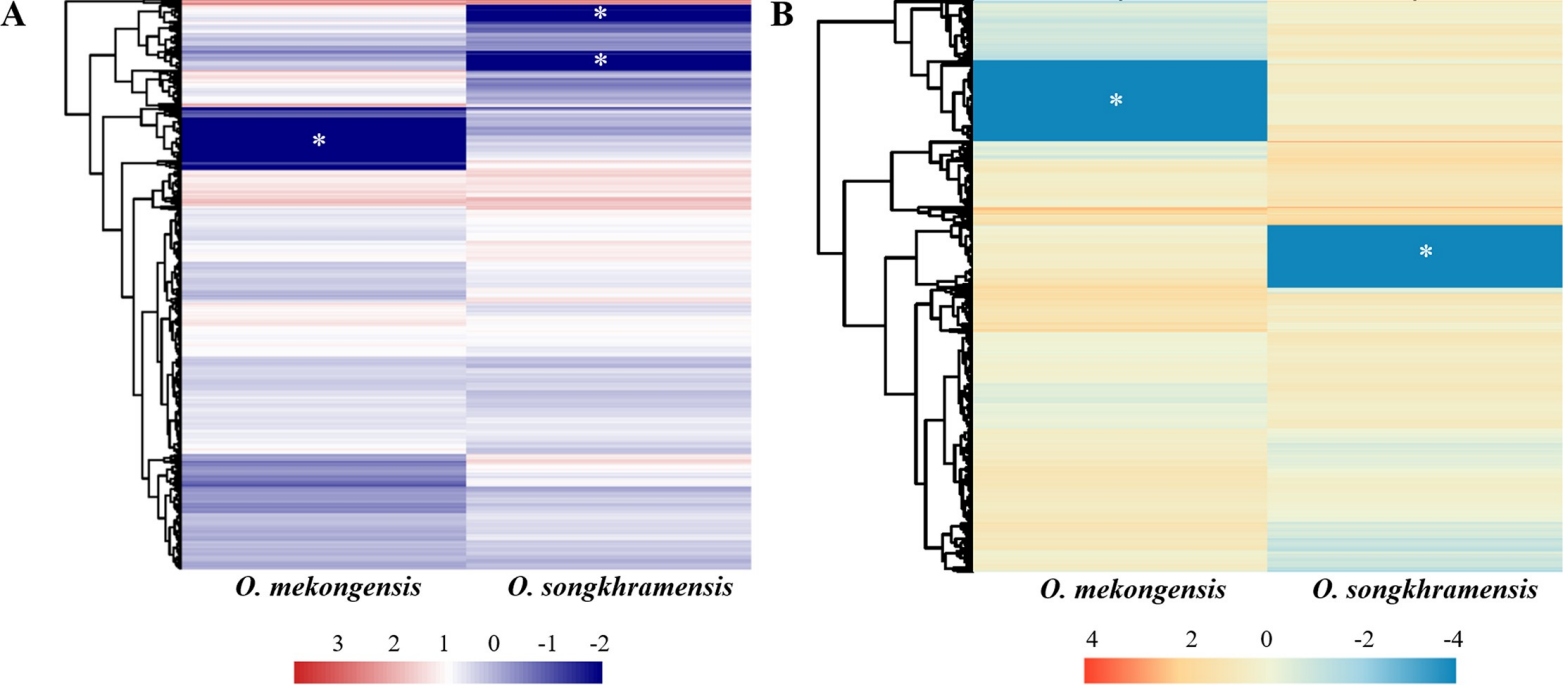
Unigenes encoding predictions of transcription factor expression (A) and differentially expressed genes (B) between *O*. *Mekongensis* and *O*. *Songkhramensis* from the heatmap representations. Asterisks indicate the substantially lower expression of gene clusters in both species.

In the transcriptomic analysis of the adult fish, the distributions of the non-redundant database (NR) annotated species of *O*. *mekongensis* and *O*. *songkhramensis* were similarly matched to *O*. *latipes* at 78.74 and 77.66%, respectively (Fig 2A and 2B). In KOG (euKaryotic Orthologous Groups), 25 functions were contained in *O*. *mekongensis* and *O*. *songkhramensis*, and “signal transduction mechanisms” and “general function prediction only” dominated in both species of medaka fish. In contrast, “coenzyme transport and metabolism” was the least represented among the gene function types in both *O*. *mekongensis* and *O*. *songkhramensis* (Fig 3A and 3B). In the gene ontology (GO) annotation, the three main categories of gene or gene products were represented, including “biological process”, “cellular component” and “molecular function”. Twenty-seven gene processes of “biological process” and twelve gene functions of “molecular function” were detected in *O*. *mekongensis* and *O*. *songkhramensis*. However, “cellular component” accounted for 19 and 16 components in *O*. *mekongensis* and *O*. *songkhramensis*, respectively. The categories “components of nucleoids”, “other organisms” and “other organism parts” were lacking in *O*. *songkhramensis*. Cellular process, cell and binding accounted for the highest gene numbers for their categories in both species (Fig 4A and 4B). In the KEGG (Kyoto Encyclopedia of Genes and Genome), the six major classes consisted of “cellular processes”, “environmental information processing”, “genetic information processing”, “human diseases”, “metabolism” and “organismal systems”. Cellular community-eukaryotes, signal transduction, folding, sorting and degradation, cancers: overview, global and overview maps and immune system showed the highest gene numbers among their classes for both *O*. *mekongensis* and *O*. *songkhramensis* (Fig 5A and 5B). In the SwissProt annotation, the unigenes of *O*. *mekongensis* approached sp|Q5T197|DCST1_HUMAN with expectation values (e-values) of 1.3e-27 and 1.0e-20 and sp|Q68F72|S15A4_XENLA with e-values of 1.2e-218 and 2.1e-55 in the SwissProt database. In *O*. *songkhramensis*, the unigenes were annotated to sp|P58875|S14L2_BOVIN with e-values of 1.3e-108 and 5.6e-169, sp|Q91WM3|U3IP2_MOUSE with an e-value of 1.6e-158, and sp|Q14416|GRM2_HUMAN with an e-value of 1.7e-148. Details of the annotation list format are provided in the S1 Table for *O*. *mekongensis* and *O*. *songkhramensis*. Regarding TF expression, one cluster showed quite low expression in *O*. *mekongensis* compared with *O*. *songkhramensis*. In contrast, two clusters showed clearly lower TF expression in *O*. *songkhramensis* than in *O*. *mekongensis* (Fig 6A). In the DEGs, there was one gene cluster of differentially expressed factors who expression quite low in *O*. *mekongensis* compared with *O*. *songkhramensis*. The low gene expression of *O*. *mekongensis* (14.58–12.46 log2-fold) consisted of the microtubule-associated protein TAU (Mapt), integral membrane protein 2B (Itm2b or Bri2), calpactin-1 light chain, interferon regulatory factor 2 binding protein 2 (Irf2bp2), heterogeneous nuclear ribonucleoprotein A1 (Hnrnpa1), microsomal glutathione S-transferase 3 (Mgst3), lysophosphatidic acid receptor 6-like (Lpar6), histone-lysine N-methyltransferase EHMT1-like, Wiskott-Aldrich syndrome protein (WASp) and 26S protease regulatory subunit 8 (Psmc5). Similarly, for the DEGs of *O*. *mekongensis*, one cluster showed obviously lower gene expression in *O*. *songkhramensis* (Fig 6B). Gene expression of *O*. *songkhramensis* was detected at low levels (16.16–12.57 log2-fold) compared with that of *O*. *mekongensis*, followed by heat shock protein family A member 8 (Hspa8), tyrosine-protein phosphatase non-receptor (Ptpn1), sister chromatid cohesion protein PDS5 homolog B (Pds5b), cyclin-dependent kinase 8 (Cdk8), TBCC domain-containing protein 1 (Tbccd1), protein disulfide-isomerase A3 (Pdia3), calpain-1, coatomer subunit alpha, junction plakoglobin-like, plakophilin 2 and apolipoprotein A-IV-like (ApoA4).

## Discussion

In this study, the populations of *O*. *mekongensis* could be divided into four groups based on the mitochondrial cytb gene. This result was consistent with reports of fish populations that examined genetic variation during succession by using the cytb gene [14, 15, 36]. In *Oryzias*, Takehana et al. [16] reported the use of the cytb gene to understand the diversity of wild Japanese medaka populations that correlated with their geographic variation in Japan. Therefore, the cytb gene might be suitable for evaluating the genetic variation of *O*. *mekongensis* populations in the northeastern region of Thailand. Moreover, this mitochondrial gene is also a potential tool to phylogenetically differentiate among *O*. *mekongensis*, *O*. *songkhramensis* and *O*. *minutillus*. It is well known that the cytb gene is one of the standard DNA barcodes for fishes [37, 38]. The cytb gene might converted to a molecular barcode and utilized in the further identification of freshwater ricefish in Thailand. Ngamniyom et al. [12] reported a molecular diversity destitution of wild Thai medaka with biogeographic variation throughout Thailand. In the present study, the wild populations of *O*. *mekongensis* showed genetic variation but the populations of *O*. *songkhramensis* did not. The physical geography of northeastern Thailand is a plateau, and the upper part forms the structure of the Sakon Nakorn Basin involving the Mekong River, Songkhram River and complexed canals [39, 40]. Thus, the genetic diversity of *O*. *mekongensis* might depend on the Mekong and Songkhram River Basins. Magtoon [8] described the natural habitat of *O*. *songkhramensis*, which mainly lives in the upper part of the Mekong River Basin in northeastern Thailand. The distribution of *O*. *songkhramensis* was not wider than that of *O*. *mekongensis*. The population of *O*. *songkhramensis* in this study was similar to the original findings for this fish population in the report of Magtoon [8]. These results suggest that the range distribution of *O*. *songkhramensis* may be narrow, which may have resulted in the single group within the *O*. *songkhramensis* population in this study.

Morphologically, *O*. *mekongensis* and *O*. *songkhramensis* have different coloured lines on their caudal fins, but there are no colored lines on the caudal fin of *O*. *minutillus* [1, 8]. Among the three species in this study, *O*. *mekongensis* was more closely related to *O*. *songkhramensis* than *O*. *minutillus* based on the cytb partial sequences. This molecular biology result is congruent with the morphology of the caudal fin, which may be considered both with and without coloured lines.

In RNA-Seq analysis, the species distributions of *O*. *mekongensis* and *O*. *songkhramensis* were closer to those of Japanese medaka than to those of the spiny chromis (*Acanthochromis polyacanthus*), barramundi (*Lates calcarifer*), and bicolour damselfish (*Stegastes partitus*). Investigators have already provided genome data for these fish species from transcriptome profiling, genome information and biosystem databases of the NCBI [41]. Moreover, the species distributions of both of the fish in this study were more similar to those of Japanese medaka than to those of Thai medaka in a previous report by Ngamniyom et al. [12]. RNA-Seq of *O*. *mekongensis* and *O*. *songkhramensis* confirmed that transcriptomic sequences of both species were conserved with medaka species close to Japanese medaka. Furthermore, the results of this study add to the molecular genetics resources for teleost fish.

In the databases of annotated genes, the KOG, GO and KEGG catalogue groups were quite similar for the gene functions of *O*. *mekongensis* and *O*. *songkhramensis*. The predominant gene functions and processes of *O*. *mekongensis* and *O*. *songkhramensis* are presented. In addition, the general gene functions of both fish species in this study were consistent with several reports of freshwater fish, such as Thai medaka, pond loach (*Misgurnus anguillicaudatus*), Yellow River scaleless carp (*Gymnocypris eckloni*) and goldfish (*Carassius auratus*) [12, 42–44]. These results suggested that the represented gene predictions of *O*. *mekongensis* and *O*. *songkhramensis* might conserve the main gene functions not only among *Oryzias* fish but also among other freshwater fish. It is well known that unigene annotations against the databases of KOG, GO and KEGG are important for understanding the candidate genes, gene functions and biological pathways in various organisms [45, 46]. The gene predictions of *O*. *mekongensis* and *O*. *songkhramensis* were dominated by “signal transduction, signal mechanism, cellular processes, cell and binding”. Therefore, these gene functional groups might be necessary for important roles in the cell biology of *O*. *mekongensis* and *O*. *songkhramensis*, as annotated by KOG, GO and KEGG. Regarding TF expression and DEGs, TFs have been investigated as an important step in gene regulatory networks [47], and DEGs are utilized as potential markers to observe different patterns of gene expression [48]. In this study, there were polymorphisms of gene expression clusters between *O*. *mekongensis* and *O*. *songkhramensis*. These findings might be species-specific gene expression patterns that differ between *O*. *mekongensis* and *O*. *songkhramensis*.

The analysis of DEGs can be performed through transcriptomic profiles to understand alterations in genes or genes in different species [49–51]. For DEG screening, the transcriptomic analyses showed that some gene expression levels were different between *O*. *mekongensis* and *O*. *songkhramensis*. One cluster of genes was highly expressed in *O*. *mekongensis* compared with *O*. *songkhramensis*. In contrast, one cluster showed higher gene expression in *O*. *songkhramensis* than in *O*. *mekongensis*.

In general pathways or functions of gene, the Mapt is microtubules associated protein regulated by phosphorylation [52], while the Itm2b plays a role of neurite outgrowth [53]. Calpactin-1 light chain regulate cytoskeletal proteins [54], and WASp is regulator of the actin cytoskeleton [55]. In vertebrates, Irf2bp2 is known as a transcriptional corepressor [56]. Hnrnpa1 is important regulation of RNA synthesis [57], but Histone-lysine N-methyltransferase EHMT1 exhibit functions in silencing of gene expression [58]. The Lpar6 is G protein-coupled receptor of lipid signaling [59], and the Mgst3 is essential gene in metabolize of endogenous and exogenous substrates [60]. In addition, Psmc5 plays the important processes for the maintenance of protein homeostasis [61].

Hspa8 functions as a chaperone in a cellular folding of translation, and Ptpn1works as a regulator of unfolding [62, 63]. It has known that Pds5b plays a role during meiosis and DNA repair associated with chromatin [64], while Cdk8 is a coactivator in the regulated transcription of genes [65]. Gonçalves et al [66] demonstrated that Tbccd1 was required for a centrosome positioning. Pdia3 played the crucial role for catalysing formation of disulfide bonds in proteins [67]. In neurons, the calpain-1is calcium-dependent cysteine protease for neurodegeneration [68]. For cytosolic protein complex, the coatomer subunit alpha binds to dilysine motifs between the endoplasmic reticulum and golgi body [69]. Plakophilin 2 is known for a role in cell–cell adhesion [70]. In major component of high density lipoprotein, ApoA4 plays an important function in lipoprotein metabolism [71]. Considering into all above mentions of gene functions and pathways, they might therefore be differences in such predominant level-specific gene expression profiles between the two fish.

To distribute the present knowledge, we provided the molecular populations of *O*. *mekongensis* and *O*. *songkhramensis* and the transcriptomic patterns of these species from Thailand in publicly available databases. These results may enhance the available genetic resources to further understand the evolution of fish in the genus *Oryzias*.

## Supporting information

S1 Table Details of the annotation list format for *O*. *Mekongensis* and *O*. *Songkhramensis*. Tables show the information from the SwissProt database for both species.
(XLS)
